# ALS or ALS mimic by neuroborreliosis—A case report

**DOI:** 10.1002/ccr3.2569

**Published:** 2019-12-11

**Authors:** Isabelle Wirsching, Nora Ort, Nurcan Üçeyler

**Affiliations:** ^1^ Department of Neurology University of Würzburg Würzburg Germany; ^2^ Present address: Charité Department of Neurology University of Berlin Berlin Germany

**Keywords:** ALS mimic, El Escorial, motor neuron disease, muscle atrophy, neuroborreliosis

## Abstract

Comprehensive investigation in motor neuron disease is vital not to miss a treatable differential diagnosis. Neuroborreliosis should be considered during an ALS work‐up. However, false‐positive CSF results do occur, and thus, results should be interpreted carefully in context of all clinical test results.

## Graphical abstract

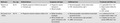

## 1 INTRODUCTION

We report a 63‐year‐old man, who developed bilateral atrophic arm paresis with preserved reflexes. Criteria for probable, laboratory‐supported ALS (El Escorial) were fulfilled. However, cerebrospinal spinal fluid analysis revealed neuroborreliosis. We give a detailed description of our patient's case and review literature on neuroborreliosis‐associated ALS mimics.

The pathophysiology of ALS is incompletely understood, and despite reports on promising first candidates,^1^, ^2^ objective diagnostic biomarkers are lacking. Hence, the diagnosis is mainly based on clinical signs and symptoms, and the results of neurophysiological studies.^3^, ^4^, ^5^, ^6^, ^7^ Since no causative treatment is available, the differentiation from other potentially treatable neurological diseases that may mimic ALS symptoms is vital. Here, we report a patient with typical manifestations of motor neuron disease and the chance finding of acute neuroborreliosis.

## 2 CASE REPORT

### 2.1 Patient history

The 63‐year‐old man was admitted for second opinion with the suspected diagnosis of a motor neuron disease. He was seen at three visits (July, August, and December 2018). The patient had been healthy until February 2018, but then developed progressive muscle weakness starting at his left proximal arm. In March 2018, he noticed ubiquitous fasciculations and generalized muscle wasting including his legs without paresis of lower extremities. In July 2018, that is, within six months, he developed paraparesis of both arms and also suffered from cramps in the shoulder girdle and hand muscles. By August 2018, the patient was severely impaired in everyday life activities. He had never experienced sensory symptoms or pain. The patient reported a tick bite in September 2017, however, without the development of an erythema migrans. Except for type II diabetes mellitus which was treated with metformin 1000 mg per day and a cervical spinal canal stenosis without signs of myelopathy, the patient did not have relevant diseases. No further regular medication was taken. The family history was uninformative with regard to neuromuscular diseases. He did not drink alcohol regularly and had stopped smoking 20 years ago.

### 2.2 Neurological examination

At his first neurological examination, in July 2018 no relevant bulbar symptoms were found except for mild fasciculations of the tongue. Facial nerve functions and swallowing were normal. Tests for motor function revealed the following paresis according to the Medical Research Council Scale (MRC; results given as right/left side): head flexion 4/5, shoulder abduction 3‐4/4−, shoulder adduction 4+/4+, shoulder internal rotation 4+/4−, shoulder external rotation 2‐3/2‐3, arm flexion 4+/4−, arm extension 4+/2‐3, arm supination 4+/1‐2, arm pronation 4+/4, hand flexion 5/5‐, hand extension 5‐/4+, finger extension 5‐/4+, finger spreading 4+/4−, bilateral deficits in finger adduction and especially finger 5, thumb abduction 4/4, thumb adduction 5/5−, thumb flexion 5/4, and thumb extension 5/4−. So, asymmetric distal and proximal atrophic arm paresis (left > right) was found while the lower extremities were spared. Muscle tone was flaccid. Muscle atrophy was seen at the shoulder girdle and fasciculations at all four extremities. Deep tendon reflexes at the upper extremities were normal, while they were reduced at the lower extremities. Babinski's sign was negative. The sensory and coordination systems were spared. On the revised ALS Functional Rating Scale (ALS‐FRS),^8^ the patient reached 43 (0‐47) points.

### 2.3 Laboratory tests

Extensive laboratory tests were normal, except for mild increase in serum creatine kinase levels (224 [0‐190] U/l). Diabetes mellitus was controlled with a current HbA1c of 7.2% (4%‐6%) and mild glucosuria and ketonuria.

### 2.4 Nerve conduction studies

There was shown a motor and axonal neuropathy without conduction blocks. Axonal loss was found with reduction of CMAP amplitudes of the left ulnar nerve (distal stimulation: 11.0 mV, proximal stimulation below ulnar sulcus: 9.2 mV, and proximal stimulation above ulnar sulcus: 8.2 mV), left median nerve (distal stimulation: 2.9 mV and proximal stimulation: 2,7 mV), and left peroneal nerve (distal stimulation: 8.0 mV, proximal stimulation below fibular head: 3.7 mV, and proximal stimulation above fibular head: 3.4 mV). Distal motor latency and nerve conduction velocity as well as sensory nerve conduction studies were normal.

### 2.5 Electromyography

**Figure 1:**
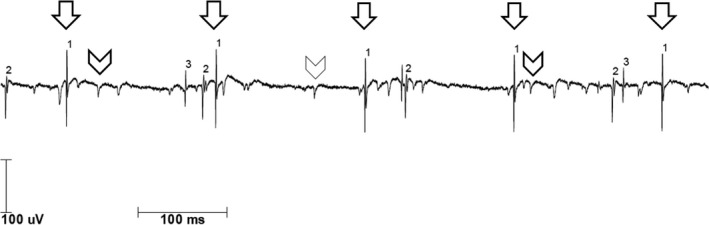
Excerpt from the electromyographic recording of the left first dorsal interosseous muscle. Fibrillations (arrows) and sporadic positive sharp waves (arrow heads) are seen

Electromyography (tongue, masseter, deltoid, interosseus dorsalis I, anterior tibial muscles, paravertebral muscles (th8 level), and femoral muscles) showed hints for chronic and acute denervation with spontaneous activity (fibrillations, positive sharp waves, and fasciculations; Figure 1). The tongue was spared.

### 2.6 Evoked potentials

Motor evoked potentials did not show signs of pyramidal impairment or an involvement of the first motor neuron when obtained from the legs and the arms.

### 2.7 Imaging results

Cranial and spinal MRI did not show any definitive pathology. However, cranial MRI showed a slight increase in signal intensity along the pyramidal tract on the proton‐weighted sequences. Additionally, the right median nerve was investigated using high‐resolution ultrasonography (18 MHz probe, transversal plane) and showed normal cross‐sectional area and echogenicity. All muscles investigated (biceps, triceps, flexor digitorum, and infra‐ and suprahyoidal muscles) ubiquitously showed deep and superficial fasciculations (Video S1). Muscles of the upper extremities were atrophic and hyperechogenic.

### 2.8 Cerebrospinal fluid analysis and infectiological laboratory

Analysis of the cerebrospinal fluid (CSF) in August 2018 revealed a pleocytosis (27 [1‐4] n/mm^3^) and increased protein (111 [0‐50] mg/dL), albumin (89.3 [0‐53] mg/dL), and lactate (2.7 [1.2‐2.1] mmol/L) levels, as well as an increased albumin ratio (18.6 [9]). It is of note that the initial lumbar puncture was accidentally traumatic and hence showed erythrocyte contamination (43.200 [0‐<1] n/mm^3^), which was first interpreted as causative for the pleocytosis. However, while virologic examination of CSF was normal, microbiological analysis surprisingly revealed positive IgM and IgG titers for antibodies against borrelia burgdorferi. Laboratory tests were performed in serum and CSF. First, an ELISA was done for screening and results were confirmed using an immunoblot. Serum tests (both with ELISA and immunoblot) were positive for IgG and negative for IgM. In line with recommended standards, the relevant antibody index was assessed, which was increased in terms of IgM antibody index 2.3 (<1.4)), while the IgG antibody index was normal. Furthermore, laboratory tests on VDRL, VZV, HSV1/2, and FSME were performed. Test results in CSF were each negative; in serum, a constellation indicative of past HSV and VZV infection was found and for FSME immunization, while results for serum VDRL were negative and therefore not further analyzed in CSF. Thus, the diagnosis of neuroborreliosis was made.

## 3 TREATMENT AND CLINICAL COURSE

Upon the microbiological CSF finding, we applied antibiotic treatment with iv ceftriaxone 2 g per day for 3 weeks. During hospitalization, no relevant improvement was observed. However, the IgM titer for antibodies against borrelia burgdorferi was negative, the IgM and IgG antibody index normal, while the IgG titer was still elevated as a sign for past infection. All other CSF parameters remained elevated, but mildly decreased: protein (89.6 [0‐50] mg/dL), albumin (66.8 [0‐53] mg/dL), lactate (2.5 [1.2‐2.1] mmol/L), and the albumin ratio (16.5 [9]).

## 4 FOLLOW‐UP

In December 2018, 11 weeks after a 4‐week rehabilitation therapy, the patient was re‐examined at our department. Unfortunately, he negated any improvement upon completion of the antibiotic and rehabilitative treatment. While relevant bulbar symptoms were still missing, he reported progressive weakness of his arms and now also of his legs. Neurological examination showed a tetraparesis with further reduction in strength of arm flexors and new paresis of toe extensors. Deep tendon reflexes were preserved and brisk even in paretic muscles and the tongue showed progressive atrophy and fasciculations. Pseudobulbar symptoms or other signs of an involvement of the upper motor neuron were missing. The ALS‐FRS had dropped to 40 (0‐47) points. Nerve conduction studies revealed a mild progression in motor, and axonal neuropathy and electromyography showed spontaneous activity now also in the biceps muscle. Deep and superficial fasciculations were still visible during HRUS including the infra‐ and suprahyoidal muscles. Motor evoked potentials elicited after cortical stimulation and recording from the arms showed mild dispersion of potentials, while central and peripheral latencies were normal.

## 5 DIFFERENTIAL DIAGNOSIS

Antibiotic treatment and subsequent rehabilitation did not improve symptoms or slow down disease progression. Hence, it is unlikely that neuroborreliosis was the main cause of symptoms in our patient. Since one year after symptom onset, next to signs of a lower motor neuron impairment, few, but definite clinical signs of an upper motor neuron impairment (preserved reflexes despite atrophic progressive paresis) and electrophysiological signs of acute and chronic denervation were found, the El Escorial criteria for probable, laboratory‐supported ALS and Awaji criteria for possible ALS were fulfilled.^4^, ^5^ Initial clinical presentation suggested the differential diagnosis of a flail arm syndrome^9^; however, fasciculations of the tongue and paresis of lower extremities developing with time suggest an early stage of ALS.

## 6 DISCUSSION

We report a patient, who developed atrophic paresis of his arm muscles over six months, initially presenting with asymmetric paresis, fasciculations of the tongue, and preserved reflexes in the clinical examination, and the incidental finding of neuroborreliosis analyzing CSF. Literature search revealed few case reports of patients with a motor neuron impairment caused by neuroborreliosis (Table 1).^10^, ^11^ While the pathophysiology remains elusive, these patients had ample clinical signs of combined spastic and atrophic tetraparesis, bulbar symptoms, and lymphogenic pleocytosis as well as microbiological evidence of neuroborreliosis in CSF analysis.^10^, ^11^

**Table 1:** Synopsis of cases described in literature on amyotrophic lateral sclerosis mimics caused by neuroborreliosis

Reference	Sex, Age	Medical history	Symptoms and signs	Additional tests	Treatment and follow‐up
Hänsel et al (1995) 11)	M, 61	Rapidly progressive bilateral arm paresis Unsteady gait	Progressive spastic tetraparesis Muscle atrophy	Electromyography: chronic denervation CSF: neuroborreliosis, IgG ↑	iv penicillin G for 3 wk Improvement within few months CSF: normal
De Cauwer et al (2009) 11	M, 76	Progressive muscle weakness within 4 wk	Bulbar dysarthria, dysphagia Mixed atrophic and spastic tetraparesis with fasciculations	Electromyography: fasciculations in proximal and distal muscles CSF: neuroborreliosis, IgG and IgM ↑ sMRI: cervical lesion	iv ceftriaxon for 4 wk Improvement within 1 wk Complete recovery incl. normal sMRI after 5 mo

Abbreviations: ALS, amyotrophic lateral sclerosis; CSF, cerebrospinal fluid; F, female; iv, intravenous; and M, male; sMRI, spinal magnetic resonance imaging.

Similarly, our patient developed progressive tetraparesis beginning at the upper limbs. Clinical findings together with nerve conduction studies indicated at least a probable, laboratory‐supported ALS. CSF analysis gave hints for neuroborreliosis though and the patient reported a tick bite in temporary context with symptom onset. However, in contrast to the cases reported in literature, our patient did not benefit from the antibiotic therapy, but showed sustained progression. The clinical course and missing effect of antibiotic treatment suggest that CSF findings were incidental and that the main diagnosis is rather ALS.

The pathomechanism of neuroborreliosis is unclear. Borrelia burgdorferi may directly impair neural structures and may mediate autoimmune processes via B‐ and T‐cell activation.^12^ A cross‐reaction between flagellin of borrelia burgdorferi and neural antigens may be of pathophysiological relevance.^12^ Electrophysiological data and few histopathological reports available mainly indicated axonal damage^13^ by borrelia burgdorferi. The outer surface protein C may act as major virulence factor.^14^

In case series of ALS in endemic areas, a correlation was found between seropositivity for borrelia burgdorferi and ALS^15^ which was, however, not confirmed in later studies (see Table 2).^16^, ^17^

**Table 2:** Small case series suggesting neuroborreliosis as a relevant differential diagnosis of motor neuron disease and large studies showing no association between neuroborreliosis and amyotrophic lateral sclerosis

Reference	Number of patients with amyotrophic lateral sclerosis	Number of patients with serological evidence of neuroborreliosis	Association between amyotrophic lateral sclerosis and neuroborreliosis
Halperin et al (1990)^15^	19	9 (ELISA and Lyme borreliosis CSF index)	Yes, “there appears to be a statistically significant association between ALS and immunoreactivity to B burgdorferi”
Waisbren et al (1987)^18^	54	4 (no further information provided)	Maybe; “it seems reasonable to find out if a patient with ALS does have B burgdorferi antibodies.”
Qureshi et al (2009)^19^	414	24 (5.8%) (ELISA) 4 (0.97%) (immunoblot)	No, it “is not likely to be either an etiological factor or of significance in the differential diagnosis of motor neuron disease.”
Visser et al (2017)^17^	491	20 (4.1%) (immunoblot) versus 58/982 (5.1%) in healthy controls	No, “this large case–control study provides evidence for a lack of association between *B burgdorferi* antibodies and ALS”

Abbreviations: ALS, amyotrophic lateral sclerosis; B., Borrelia; ELISA, enzyme‐linked immunosorbent assay.

It is controversially discussed, if microbiological tests for borrelia burgdorferi in CSF should be included in routine work‐up of patients with assumed motor neuron disease.^17^, ^18^ Given the lack of objective biomarkers and relatively low sensitivity of the currently used diagnostic criteria,^3^, ^6^, ^7^ it is vital to exclude potentially treatable diseases in the differential diagnostic work‐up of all patients not to miss seldom, but treatable differential diagnoses^20^ such as neuroborreliosis. Although rare, patient cases as described in Table 1 strongly advocate this notion. Therefore, Neuroborreliosis should be considered during an ALS work‐up. However, false‐positive CSF results do occur. For example, one systematic review on twenty neuroborreliosis case‐control studies investigated accuracy of serological tests. Their overall sensitivity was 77% (95%, confidence interval 67% to 85%) and specificity 93% (95%, confidence interval 88% to 96%).^21^ Thus, results of CSF in ALS work‐up should be interpreted carefully in context of all clinical test results. Further studies investigating large series with neuroborreliosis to identify specific biomarkers applicable in differential diagnosis with ALS may be useful.

## CONFLICT OF INTEREST

None of the authors has any conflict of interest to disclose.

## AUTHORS CONTRIBUTION

IW: involved in clinical examination, collected data, and reviewed and approved the final manuscript. NO: involved in clinical examination, collected data, and reviewed and approved the final manuscript NÜ: involved in clinical examination, collected data, and reviewed and approved the final manuscript.
